# Disease Sequences High-Accuracy Alignment Based on the Precision Medicine

**DOI:** 10.1155/2018/1718046

**Published:** 2018-02-22

**Authors:** ManZhi Li, HaiXia Long, HongTao Wang, HaiYan Fu, Dong Xu, YouJian Shen, YuHua Yao, Bo Liao

**Affiliations:** ^1^School of Mathematics and Statistics, Hainan Normal University, Haikou, Hainan 571158, China; ^2^School of Information Science Technology, Hainan Normal University, Haikou, Hainan 571158, China

## Abstract

High-accuracy alignment of sequences with disease information contributes to disease treatment and prevention. The results of multiple sequence alignment depend on the parameters of the objective function, including gap open penalties (GOP), gap extension penalties (GEP), and substitution matrix (SM). Firstly, the theory parameter formulas relating to GOP, GAP, and SM are inferred, combining unaligned sequence length, number, and identity. Secondly, we tested the rationality of the theory parameter formulas, with experiment on the ClustalW and MAFFT program. In addition, we obtained a group of MAFFT program parameters according to the formulas proposed. The results of all experiments show that the SPS (sum-of-pair score) obtained from theory parameters is better than the SPS obtained from the default parameters of ClustalW and MAFFT. In both theory and practice, our method to determine the parameters is feasible and efficient. These can provide high-accuracy alignment results for precision medicine.

## 1. Introduction

In 2015, US President Barack Obama stated his intention to fund a United States national “Precision Medicine Initiative” [1, 2]. A short-term goal of the Precision Medicine Initiative is to expand cancer genomics to develop better prevention and treatment methods. With the explosive growth of medical data, the complexity of disease, and the demand of personalized medicine, the research results of genome sequencing are changing the process of disease treatment. Multiple sequence alignment (MSA) is more and more important.

Multiple sequence alignment (MSA) has wide applications in sequence analysis, gene recognition, protein structure prediction, and reconstructing the phylogenetic tree [3]. Notredame [4] stated that the most modern programs for constructing MSA consist of two components: (1) an objective function to assess the quality of candidate alignment and (2) an optimization procedure for identifying the highest scoring alignment with respect to the chosen objective function. Currently, MSA has three main objective functions: (1) the sum-of-pairs score function (SPS), (2) the consensus function, and (3) the tree function. The SPS function is the most commonly used objective function, and its parameters include substitution matrix and gap opening penalties (GOP) and gap extending penalties (GEP).

The parameters of the objective function have generated many discussions on how to obtain optimal parameters. Thompson et al. [5] determined that substitution matrices vary at different alignment stages according to the divergence of sequences to be aligned. Residue-specific gap penalties and gap penalties in hydrophilic regions, which have been locally reduced, can cause new gaps to appear in potential loop regions rather than in a regular secondary structure. Reese and Pearson [6] discussed the relational formula between the PAM distance and PAM matrix as well as the gap penalty. Madhusudhan et al. [7] proposed the variable penalty formula according the structure of sequence based on dynamic programming. However, these formulas are not widely used. Gondro and Kinghorn [8] indicated that gap penalty parameters were determined by experience. At present, it is no theoretical framework to determine the optimum parameters. The current parameters pertaining to the objective function in most literature are empirical values which are independently associated with the sequences [9]. BALiBASE is a database of manually refined multiple sequence alignments [10] and is usually used to test performance of MSA method [11].

Many open source online alignment tools are available that can align hundreds of thousands of sequences in hours. These include CLUSTAL Omega, T-COFFEE, and MAFFT, [5, 12–14] and often become the primary source of sequence alignment solution. However, these MSA tool results strongly depend on the gap penalty and substitution matrix. Different parameter combinations can obtain different MSA results. The majority of users use a single default parameter when applying these alignment tools, but the results are not the best. Moreover, an effective methodology has not yet been developed to directly determine an MSA optimal parameter, which means current online tools cannot guarantee the best solution. However, when compared with other MSA alignment tools, MAFFT has the advantage of simple input parameters and obtains better results than the other tools [12, 13]. This paper uses MAFFT as the basic experimental tool to verify the accuracy of the original formulas presented herein as they relate to the substitution matrix and the gap penalty.

## 2. Sum-of-Pairs (SP) Objective Function

The sum-of-pairs (SP) function is commonly used as an objective function for MSA and is derived as

(1) $$\begin{array}{c} score=\sum Residue-\sum penalty, \end{array}$$

where the score is >0. When the score is higher, the accuracy of MSA is higher [15]. ∑Residue > 0 represents the total score of amino acid residues in the alignment sequence. ∑penalty is the total penalty score due to inserting gap and ∑penalty > 0.

∑Residue is calculated as

(2) $$\begin{array}{c} \sum Residue=\overset{L}{\underset{h=1}{\sum }}\overset{k-1}{\underset{i=1}{\sum }}\overset{k}{\underset{j=i+1}{\sum }}\mathrm{Cost}\left(\;S_{i},S_{j}\;\right), \end{array}$$

where *S*_*ih*_ is the *h* residue of the *i* sequence,* L* is the length of the aligned sequences, and *k* is the number of the sequences.

(3) $$\begin{array}{c} \mathrm{Cost}\left(\;S_{i},S_{j}\;\right)=\left\{\begin{array}{cc} S_{aa}=Score\left(a,a\right) & if\;a=a\;\;\left(residues\;\;are\;matched\right)\\ S_{ab}=Score\left(a,b\right) & if\;a\neq \text{“}-\text{”},\;\;b\neq \text{“}-\text{” }\left(residues\;are\;mismatched\right)\\ S_{a-}=Score\left(a,-\right)=0 & if\;a\neq \text{“}-\text{” }\left(residue\;and\;gap\right). \end{array}\right. \end{array}$$

Cost is computed by a substitution matrix. Currently, two main kinds of substitution matrices are available: PAM and BLOSUM. The BLOSUM series applies to this research. In substitution matrices, *S*_*aa*_ are different from each other. When the residues are mismatched, *S*_*ab*_ are also different from each other. But, in the process of simplifying the calculation, we need to use a precise and representative numerical value to represent the characteristics of the matrix. The average value can be a good characteristic representing a group of different data. Therefore, using the average value mean(*S*_*aa*_) of *S*_*aa*_ represents the match of the matrix and using an average value mean(*S*_*ab*_) of *S*_*ab*_ represents the mismatch of the matrix.

The calculation of ∑penalty is divided into two categories: linear penalty and affine penalty. Linear penalty penalizes the same score for each gap. Affine penalty is commonly used because it is biologically meaningful [16–18]. The gap is divided into two types: gap open penalty (GOP) and gap extension penalty (GEP), so the affine penalty formula is given as

(4) $$\begin{array}{c} \sum penalty=N_{GOP}\cdot GOP+N_{GEP}\cdot GEP, \end{array}$$

where *N*_GOP_ is the number of GOP, *N*_GEP_ is the number of GEP, and GOP > GEP.

## 3. The Theory Parameters Determination of SP Function for MSA

*Symbol Description*. The number of unaligned sequences is *m*. The length of the longest sequence is len_max_. The length of the shortest sequence is len_min_. The mean identity is iden. The number of amino acid residues matched is num_match_ = (*m*(*m* − 1)/2) · len_min_ · iden.  After alignment, the number of gaps inserted into each sequence is num_gap_.

**Figure 1:**
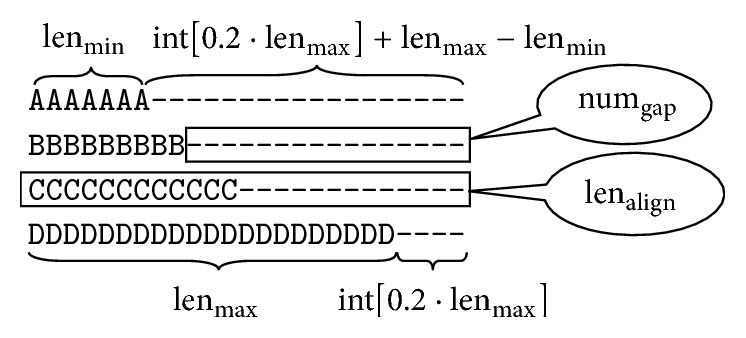
The relationship between the sequence length and the number of gaps.

Table 1 summarizes the ratio of the longest sequence and the number of gaps inserted into the sequence of each data set in BAliBASE 2.0 and BAliBASE 3.0. It shows that the number of gaps in the longest sequence is not more than 0.2 times the length of the longest sequence. That is, the number of gaps in each sequence is num_gap_ ≤ int (0.2 · len_max_) + len_max_ − len_min_, and int is the rounding function.  Figure 1 shows how the sequence length and the number of gaps num_gap_ are related.

Figure 1 is an example. If len_align_ = 25, len_max_ = 21, and len_min_ = 7, the number of gaps inserted into the longest sequence is num_gap_ = len_align_ − len_max_ = 25 − 21 = 4, and the ratio between the sequence and gaps is ratio = (len_align_ − len_max_)/len_max_ = 4/21 = 0.19. The number of gaps in the sequence is num_gap_ ≤  int [0.2 · len_max_]. The number of gaps inserting the shortest sequence is num_gap_ = len_align_ − len_min_ = 25 − 7 = 18, and the number of gaps in sequence is num_gap_ ≤ int[0.2 · len_max_] + len_max_ − len_min_. The number of gaps in other sequences is num_gap_ ≤ int[0.2 · len_max_] + len_max_ − len_min_.

**Figure 2:**
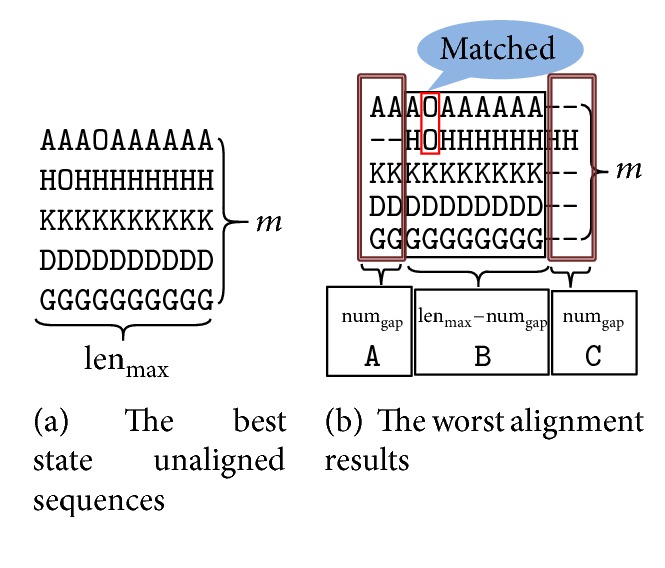
Unalignment and alignment.

The following parameter formulas are inferred according to information obtained from Figure 2.  Figure 2(a) has the best state unaligned sequence. Each sequence has the same length and no gaps. The longest length of any unaligned sequence is 10, so the number of gaps inserted can go up to 2.  Figure 2(b) shows the worst alignment results (inserting maximum gap and minimum matching). If the score of Figure 2(b) is higher than the score of Figure 2(a), the parameters of the objective function meet all cases of alignment, because the situation in Figure 2 is the worst alignment.

### 3.1. Substitution Matrix Theory Formula

According to (1), the SP score of unaligned sequences is

(5) scorebegin=∑Residue−∑penalty=mm−12·lenmax·Sab

and according to (1) and Figure 2(b), the following equations can be obtained:

(6) scoreend=score A+score B+score C,score A=α·m−1m−22·numgap·Sab,score B=mm−12lenmax−numgap−nummatch·Sab+β·nummatch·Saa,score C=∑penalty.

So, the SP score of the aligned sequences is

(7) scoreend=mm−12·lenmax−numgap−nummatch+α·m−1m−22·numgap·Sab+β·nummatch·Saa−∑penalty.

In theory, the alignment score must be greater than the unaligned sequence score,

(8) $$\begin{array}{c} score_{begin}\leq score_{end}. \end{array}$$

That is,

(9) mm−12·lenmax·Sab≤mm−12·lenmax−numgap−nummatch+α·m−1m−22·numgap·Sab+β·nummatch·Saa−∑penalty.

Equation (9) can be simplified as

(10) Saa≥αm−2α−m1−m2β·numgapnummatch+1β·Sab.

The formula of the substitution matrix is shown in (10), which can be simplified as

(11) $$\begin{array}{c} reference\geq calc. \end{array}$$

The rationality of the substitution matrix can be judged according to (11).

### 3.2. GOP and GEP Theory Formulas

Based on the affine penalty, num_gap_ is the number of gaps of each sequence; let us suppose that the number of gaps in each sequence is *λ* times as the number of GOP, so *N*_GOP_ = *m* · (1/*λ*) · num_gap_ and *N*_GEP_ = *m* · (1 − 1/*λ*) · num_gap_. Because GOP > GEP, we accept that GOP = *n* · GEP, where *λ*, *n* is the positive integer, so

(12) ∑penalty=NGOP·GOP+NGEP·GEP=n+λ−1nλ·m·numgap·GOP.

According to (12), (9) can be expressed as follows:

(13) αm−2α−mm−12·numgap·Sab+nummatch·βSaa−Sab≥∑penalty⟹GOP≤αm−2α−mm−12numgapSab+nummatchβSaa−Sab·nλmn+λ−1·numgap.

Equation (13) is the upper limit of GOP and the lower limit is GOP > 0.

If the upper limit of GOP is multiplied by weight coefficient *ω* and 0 < *ω* < 1, the estimation formula of GOP is

(14) GOP=ω·αm−2α−mm−12numgapSab+nummatchβSaa−Sab·nλmn+λ−1·numgap,

where num_match_ = (*m*(*m* − 1)/2) · len_min_ · iden, num_gap_ = int(0.2 · len_max_) + len_max_ − len_min_, and int is a rounding function. len_min_ is the length of the shortest sequence in the unaligned sets, and iden is the mean identity of unaligned sets.

The estimation formula of GEP is

(15) $$\begin{array}{c} GEP=\frac{GOP}{n}. \end{array}$$

The optimal value of each weight coefficients *λ*, *n*, *ω*, *α*, and *β* in (14) and (15) can be obtained through the following experiments.

## 4. Simulation and Results

In order to test the rationality of the parameter formulas and determine the optimal value of each weight coefficient, we designed the following experiments on the BAliBASE 2.0 and BAliBASE 3.0.

### 4.1. Experiment Setting

BAliBASE version 2.0 [10] is an improved version, extended from version 1 with 167 reference alignments to over 2100 sequences, which also features eight reference sets. Because all the reference alignments of BAliBASE are aligned by the manual, it often used to test algorithms [19–21]. Because our study is based on the global SP function, in this article, we used 113 reference alignments in References 1–3 as test objects. BAliBASE version 3.0 has the most widely used multiple alignment benchmark. The database contains 218 multiple protein sequence alignments, which have been divided into five reference sets. The first reference set includes equidistant sequences, whose identity is less than 20% (RV11) or between 20 and 40% (RV12) [22]. Other references have no similarity information. Because the formulas proposed in this paper need similarity of sequences, BAliBASE 2.0 and BAliBASE 3.0 (RV11 and RV12) were both used to establish data sets.

SPS (sum-of-pair score) works as an objective function, which can determine score increases if sequences are correctly aligned. If the SPS is higher, the results of alignment are close to the reference alignment and can be even better than the reference alignment [20]. To test the rationality of presented formulas and to determine the optimal parameters combination of MSA tools, the most popular alignment program, MAFFT [16], is used in this research. The alignment results are obtained through the Perl programming language. The MAFFT program has some advantages: (1) the number of MAFFT program parameters is less and is easy to control, using only substitution matrices, GOP and GEP, (2) through Perl, the MAFFT program can batch align, and (3) alignment accuracy is for the most part better than CW, MUSCULE, and TCOFFEE.

In our experiment, 1 ≤ GOP ≤ 20,0 ≤ GEP ≤ GOP/2. The GOP step is 1, the GEP step is 0.2, and the substitution matrices are BLOSUM30, BLOSUM45, and BLOSUM62. For each group of sequences, through batch processing, the number of alignment results is 1,590 because there are 1,590 different combined parameter patterns.

### 4.2. Experiment Results

#### 4.2.1. The Verification of Substitution Matrix Formula

**Figure 3:**
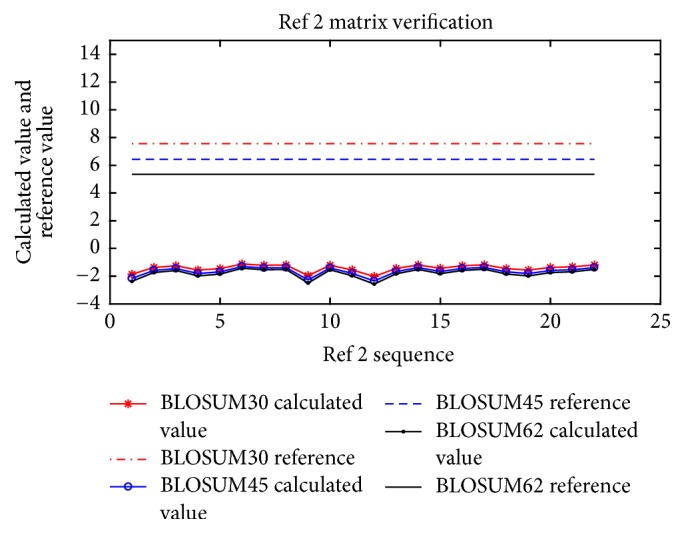
The results of the verification of substitution matrix (11).

This section shows how the rationality of the substitution matrix was established (see (11)).  Figure 3 illustrates the calculated value and reference value of each of the three substitution matrices for Reference 2 (note: the other figures are similar to Figure 3). According to (11), when the reference value is greater than the reference value, the substitution matrix is rationality. It is shown that BLOSUM30, BLOSUM45, and BLOSUM62 meet the requirements of all sequences.

Table 2 lists the number of sequences meeting the substitution matrix sequence requirements (see (11)). It is shown that three BLOSUM substitution matrices meet all the sequences for References 1–3.

#### 4.2.2. The Verification of Gap Penalty Formulas

**Figure 4:**
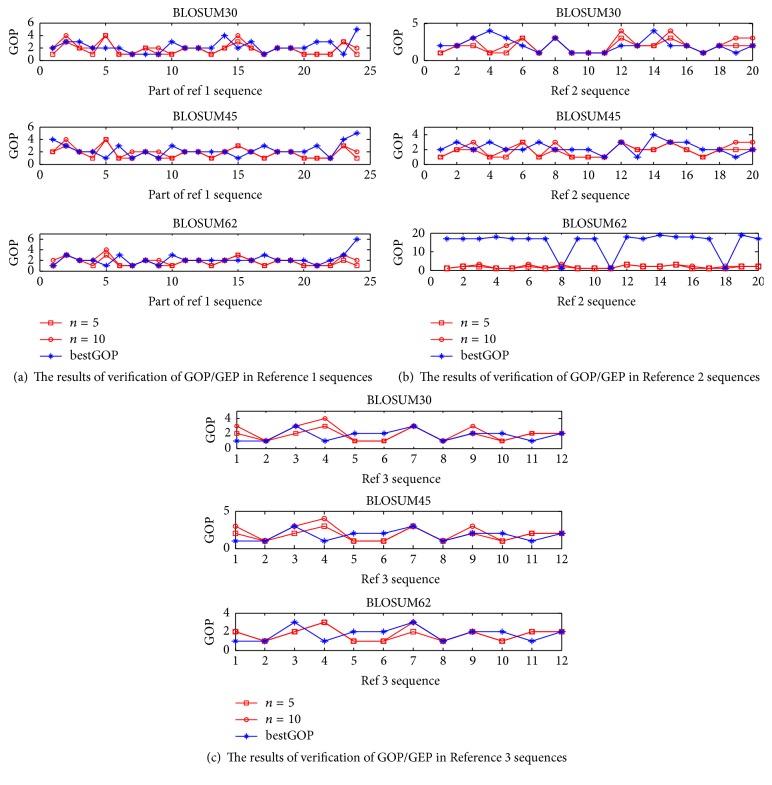
The results of verification of GOP/GEP in (14) and (15).

Based on the SPS and MAFFT program (MAFFT-7.220-WIN64 version), we tested the rationality of (14) and (15). The optimum of GOP corresponded to the maximal SPS illustrated in Figure 4. From Figure 4, we can conclude the following: the GOP theory values inferred from (14) and (15) almost coincide with the optimal of GOP, so (14) can calculate the optimal value of GOP.

Table 3 statistics show the number of sequences in Reference 1 (Test 2), which meet the theory parameter requirements corresponding to SPS, which are greater than the default parameters corresponding to SPS. In Test 2, there are 24 sequences. Table 3 shows that when *λ* = 3, *α* = 0.2, *β* = 0.9, and *n* = 5, the number of sequences is greater than *λ* = 3, *α* = 0.2, *β* = 0.9, and *n* = 10. The best result is indicated in Blosum45, num 19, with an SPS of 0.8003 (in Table 3 set in bold face font). For Test 2 sequence sets, *λ* = 3, *n* = 5 is relatively rational and corresponds to *ω* = 0.05. The other sequence sets can also obtain the value of *n*, *λ*, *ω*, *α*, and *β*, which are listed in Table 4.

#### 4.2.3. Finding Optimal Value of Other Parameters in Derivation Formula

From the aforementioned experiments, we can determine the substitution matrix and *n*, *λ*, and *ω* in (14). The other parameters are related to the sequences where *λ* is the ratio of GOP and num_gap_, and num_gap_ = int(0.2 · len_max_) + len_max_ − len_min_. The number of GOP is limited and it will not increase too much, while the distribution of GEP is more concentrated. These parameters are more consistent with the biological characteristics of multiple sequence alignment.

Optimal parameters and the SPS value are listed in Table 4. The optimal value of weight coefficient in our proposed formula is located in Table 4. Using a weight coefficient, we can obtain the optimal of GOP, GEP, and MATRIX parameters. The number of sequences corresponding to SPS is also listed in Table 4.

**Figure 5:**
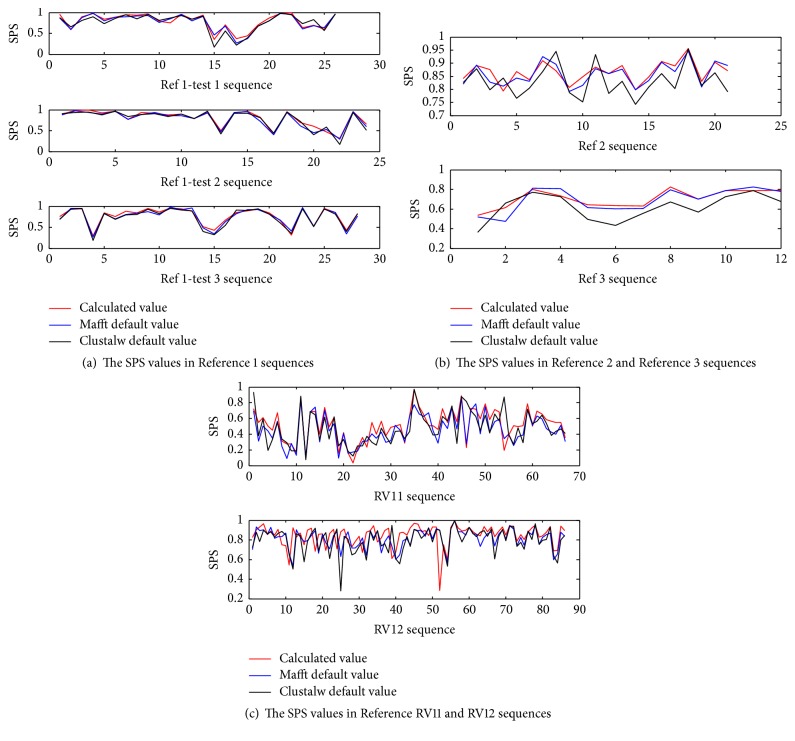
The SPS values are from MAFFT theory parameters, MAFFT default parameters, and CLUSTALW default parameter.

Figure 5 shows that, for each SPS value sequence obtained from theory parameters, we inferred default parameters of MAFFT (MAFFT-7.220-WIN64 version) and CLUSTALW (CLUSTALW-2.1-WIN version). The SPS obtained by the MAFFT program are better than the CLUSTALW program on the default parameters. So we chose the MAFFT program as our test method. The SPS obtained by our theory parameters were better than the default parameters of MAFFT and CLUSTALW. Thus, the theory parameters we propose can optimize the results of MSA.

Table 5 shows the SPS mean values of References 1–3 sequences of BAliBASE 2.0 and RV11/RV12 of BAliBASE 3.0. The alignment sequences obtained from MAFFT default parameters, CLUSTALW default parameters, and MAFFT theory parameters are those proposed in this study. It is shown that SPS values obtained by MAFFT default parameters are better than SPS values obtained by CLUSTALW default parameters. The SPS values obtained using our theory parameters are the best. So, the theory parameters optimized the results of MSA.

## 5. Conclusions

This paper clearly shows that the parameters of MSA tools influence MSA results. These parameters not only include substitution matrices, GOP, and GEP but also include the length, number, and identity of sequences. Our goal was to find a group of combined optimal parameters. Based on the SP function, we established a series of formulas which can determine the value of substitution, GOP, and GEP. In order to test the rationality of the formulas, our experiments were conducted in the MAFFT program base or in the BAliBASE 2.0 and BAliBASE 3.0 (RV11 and RV12) database. Moreover, we obtained the optimal value of the substitution matrices, GOP and GEP, and these values proved to be better than the default values of the MAFFT program. After the theory analysis and experimental analysis, we can conclude that the proposed method can effectively solve the MSA parameter problems and improve MSA accuracy, which can provide more accuracy information for precision medicine in disease analysis and prediction.

## Figures and Tables

**Table 1:** Ratio of the longest sequence and the number of gaps inserted into the sequence.

	BALIBASE 2.0					BALIBASE 3.0	
Data set	Test 1	Test 2	Test 3	Ref 2	Ref 3	RV11	RV12
Mean (ratio)	0.0769	0.0764	0.0744	0.1439	0.1612	0.1938	0.0784

**Table 2:** The number of sequences meeting the substitution matrix requirements (see (11)).

	Sequence number	Reference alignment number	BLOSUM30 qualified number (rate)	BLOSUM45 qualified number (rate)	BLOSUM62 qualified number (rate)
Reference 1	4-5	78	78 (100%)	78 (100%)	78 (100%)
Reference 2	14–19	22	22 (100%)	22 (100%)	22 (100%)
Reference 3	> 20	12	12 (100%)	12 (100%)	12 (100%)

**Table 3:** Determination of the value of *n*, *λ*, *ω*, *α*, *β*.

*n* = 5, *λ* = 3, *α* = 0.2, *β* = 0.9	BLOSUM30		BLOSUM45		BLOSUM62	
*ω*	num	SPS	num	SPS	num	SPS
0.01	7	0.7586	11	0.7768	9	0.7697
0.02	9	0.761	10	0.7769	8	0.7692
0.03	10	0.7643	11	0.7795	10	0.7703
0.04	12	0.782	15	0.7886	12	0.7745
0.05	14	0.7843	**19**	**0.8003**	15	0.7846
0.06	14	0.7805	17	0.7924	17	0.7864
0.07	14	0.7767	16	0.7896	16	0.786
0.08	14	0.7728	16	0.784	14	0.7821
0.09	14	0.7668	15	0.7804	14	0.7777
0.1	14	0.764	15	0.78	15	0.7826
0.01	7	0.7586	11	0.7768	9	0.7697
0.02	9	0.7614	10	0.7769	9	0.77
0.03	10	0.7681	12	0.7818	12	0.7744
0.04	15	0.7874	15	0.7877	13	0.7858
0.05	13	0.7783	15	0.79	16	0.7918
0.06	13	0.7736	14	0.7845	14	0.7859
0.07	13	0.7732	14	0.7781	12	0.7819
0.08	13	0.7709	16	0.7846	12	0.7713
0.09	14	0.7779	15	0.7781	13	0.7751
0.1	14	0.7731	15	0.7795	14	0.7779

**Table 4:** Optimal GOP/GEP/matrix.

Sequence set	Ref 1-test 1	Ref 1-test 2	Ref 1-test 3	Ref 2	Ref 3
Sequence row	4-5	4-5	4-5	14–19	>20
Sequence length (bp)	<100	100–300	>300	50–600	60–600
*ω*	0.03	0.05	0.08	0.02	0.02
*n*	5	5	10	10	10
Matrix	BLOSUM45	BLOSUM45	BLOSUM62	BLOSUM45	BLOSUM45
*λ*	3				
*α*	0.2				
*β*	0.9				

**Table 5:** SPS mean value.

	BaliBASE 2.0					BaliBASE 3.0	
Data set	Ref 1 (test 1)	Ref 1 (test 2)	Ref 1 (test 3)	Ref 2	Ref 3	RV11	RV12
MAFFT default parameters	0.7749	0.7743	0.7460	0.8584	0.6938	0.4582	0.8142
CW default parameters	0.7614	0.7732	0.7340	0.8311	0.6189	0.4758	0.7966
MAFFT theory parameters	0.7918	0.8003	0.7652	0.8655	0.7073	0.5183	0.8449
